# Disparities in Opioid Use for Pain Control Among Older Adults: The Role of Social Factors

**DOI:** 10.1093/geroni/igaa057.3046

**Published:** 2020-12-16

**Authors:** Taylor Jansen

**Affiliations:** University of Massachusetts Boston, Boston, Massachusetts, United States

## Abstract

Over 50% of older adults (65+ years old) suffer from pain, and an estimated 25% of older adults use prescription opioids to treat their pain. Older adults are physiologically vulnerable to the effects of opioids; yet, they are prescribed more than all other age groups. This study used the Health and Retirement Study 2016 Core dataset (N=3,916) to analyze the moderation effect of social support on the association between pain and prescription opioid use in people aged 65+ using logistical regression analysis. Results show that older adults with severe pain were more likely to use prescription opioids (OR= 4.84) after considering covariates. Higher perceived social support was associated with higher likelihood of prescription opioid use for severe pain (OR=1.53). Older adults are at greater risk of pain and social isolation compared to younger age groups, making them a vulnerable group to consider as policy makers tackle this nationwide epidemic.

